# Supplementary figures and images for: Cell wall O-acetyl and methyl esterification patterns of leaves reflected in atmospheric emission signatures of acetic acid and methanol
Source: PLoS One. 2020 May 20;15(5):e0227591. doi: 10.1371/journal.pone.0227591 (PMC7239448; doi:10.1371/journal.pone.0227591)

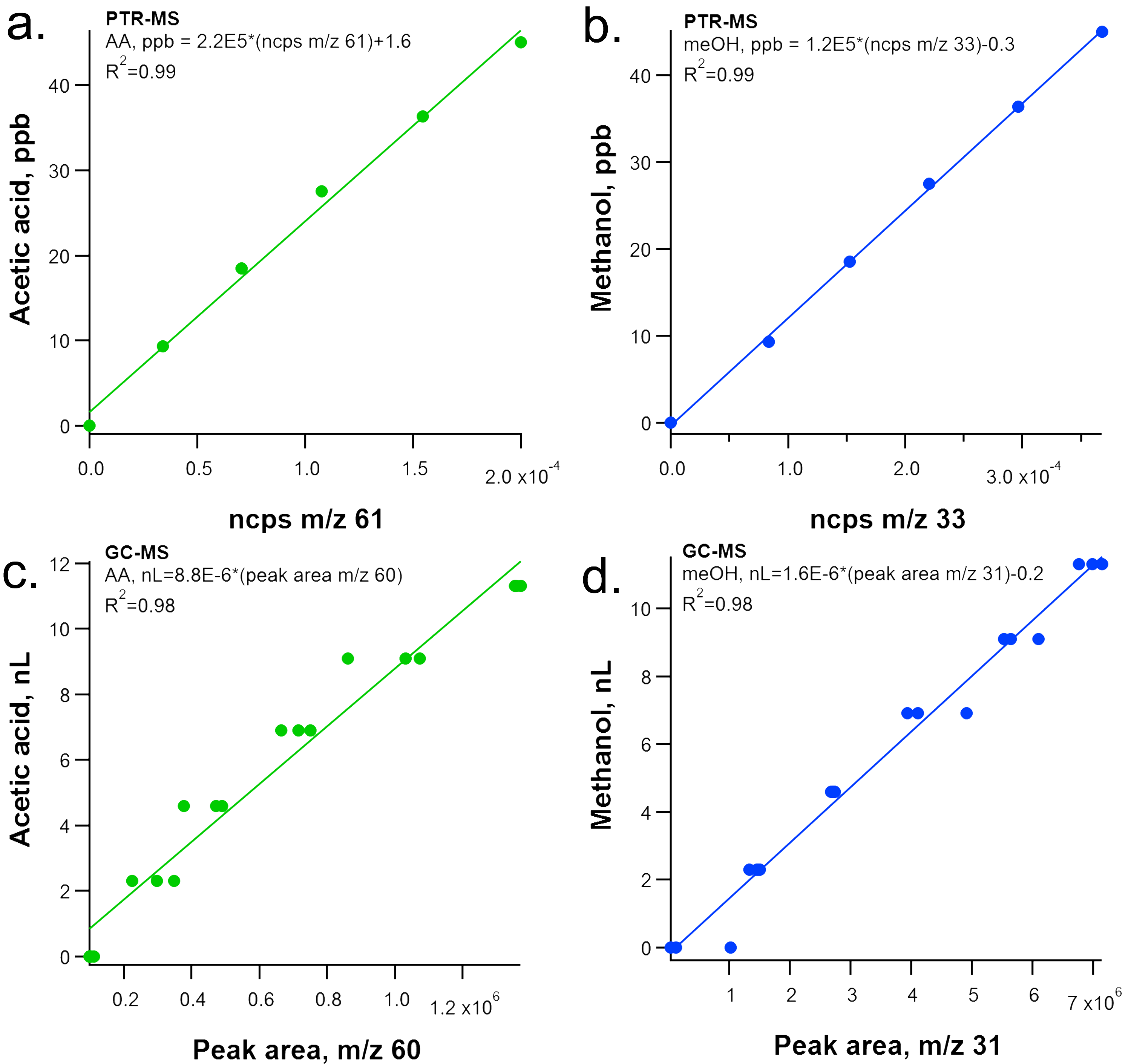

Supplement: S1 Fig — Example linear calibration responses for PTR-MS (A-B) and online GC-MS (C-D) to a primary gas-phase standard of acetic acid (AA) and methanol (meOH) on 22 June 2019. (TIF) [file pone.0227591.s001.tif]
