# Supplementary material for: Cell wall O-acetyl and methyl esterification patterns of leaves reflected in atmospheric emission signatures of acetic acid and methanol
Source: PLoS One. 2020 May 20;15(5):e0227591. doi: 10.1371/journal.pone.0227591 (PMC7239448; doi:10.1371/journal.pone.0227591)
Supplement: S1 Dataset — (DOCX) [file pone.0227591.s002.docx]

Supporting material consisting of the raw experimental data files collected and analyzed in this study are available in electronic form free of charge accessed through Mendeley Data (http://dx.doi.org/10.17632/7bdwbwy6wn.1). The supplementary data (Size: 169 MB) includes raw meOH and AA emission data obtained from the PTR-MS and online TD-GC-MS as well as raw colorimetric assay data for leaf bulk methyl and *O*-acetyl ester content organized as follows:

**Volatile emission folder:**

- Real-time time meOH and AA emission data during leaf desiccation experiments (PTR-MS)
- Near real-time time meOH and AA emission data during leaf desiccation experiments (online GC-MS)

**Cell wall esterification folder:**

- Raw absorbance data and derived total methyl ester content of AIR samples (methyl ester assays)
- Raw absorbance data and derived total acetate ester content of AIR samples (acetate ester assays)
- Derived methyl and acetate ester content of AIR samples

**Summary data:**

- A spreadsheet containing averages of the emissions and cell wall ester data used in the study
